# Nutritional risk, functional decline, and symptom burden in lung cancer: a study based on PG-SGA scores and biochemical data

**DOI:** 10.3389/fnut.2025.1545177

**Published:** 2025-04-03

**Authors:** Shasha Shen, Shiyun Xing, Tingting Shen, Li Niu, Xiaojing Tian, Hu Ma, Xiaoxia Gou

**Affiliations:** ^1^Department of Head and Neck Oncology, The Second Affiliated Hospital of Zunyi Medical University, Zunyi, Guizhou, China; ^2^Department of Thoracic Oncology, The Second Affiliated Hospital of Zunyi Medical University, Zunyi, Guizhou, China; ^3^Department of Nephrology, The Affiliated Hospital of Zunyi Medical University, Zunyi, Guizhou, China; ^4^Department of Anesthesiology, The Second Affiliated Hospital of Zunyi Medical University, Zunyi, Guizhou, China

**Keywords:** lung cancer, nutritional risk, nutritional status, quality of life, PG-SGA, functional status

## Abstract

**Objective:**

This study aims to investigate the relationship between nutritional status and quality of life (QOL) in patients with lung cancer, analyze the clinical application of nutritional support, and explore its association with biochemical markers and physical function.

**Methods:**

A total of 270 hospitalized lung cancer patients were enrolled. Demographic characteristics, cancer staging, and treatment details were collected. Nutritional and functional status were assessed using the Patient-Generated Subjective Global Assessment (PG-SGA), Nutritional Risk Screening 2002 (NRS-2002), and Karnofsky Performance Status (KPS) scores. Quality of life was evaluated with the EORTC QLQ-C30 questionnaire. The relationships between nutritional status, blood biochemical markers, body composition, and quality of life were analyzed.

**Results:**

Among the 270 hospitalized lung cancer patients analyzed, 74.81% were male, and 80.74% were aged over 65 years. PG-SGA scores indicated that 38.89% of patients were at high nutritional risk (PG-SGA ≥ 9), and 77.04% had not received nutritional support. PG-SGA scores were significantly correlated with several biochemical indicators (e.g., prealbumin, total bilirubin, alanine aminotransferase, and lymphocyte count) and nutritional parameters (e.g., NRS-2002, KPS scores, body weight, and mid-upper arm circumference). Patients with higher PG-SGA scores had significantly lower scores in physical functioning, role functioning, emotional functioning, and social functioning, alongside more severe symptoms such as fatigue, nausea, and pain. Further analysis revealed a negative correlation between PG-SGA scores and overall health status (*r* = −0.687, *p* < 0.001) and positive correlations with symptoms such as fatigue, nausea, pain, and insomnia (*r* > 0.5, *p* < 0.001).

**Conclusion:**

Nutritional status significantly impacts the quality of life in patients with lung cancer. PG-SGA scores are strongly associated with patients’ functional abilities and symptom burden. Despite the low utilization of nutritional support, particularly in high-risk groups, improving nutritional interventions may effectively enhance functional status and quality of life in these patients.

## Introduction

Lung cancer, as one of the most common and deadliest cancers globally, has become a pressing concern in the field of public health (1, 2). Despite advances in early diagnosis and therapeutic strategies, the quality of life (QOL) of lung cancer patients remains a significant challenge (3–6). This is primarily because most patients are diagnosed at advanced stages and experience multiple complications during treatment. Many lung cancer patients not only face the progression of the tumor itself but also contend with malnutrition, functional impairment, and symptom burden, all of which collectively impact their overall quality of life (7). In recent years, the importance of nutritional status in cancer treatment has gained increasing recognition. Adequate nutrition not only enhances patients’ physical function and immunity but also improves treatment tolerance, reduces complications, and elevates quality of life (8, 9). However, lung cancer patients often face significant nutritional risks during treatment, likely due to advanced disease stages, treatment modalities, and overall health conditions (10). Consequently, assessing the nutritional status of lung cancer patients is critical for developing personalized treatment strategies and improving their quality of life. The Patient-Generated Subjective Global Assessment (PG-SGA) is a widely used clinical tool for nutritional assessment and has been proven effective in evaluating nutritional risk and functional status in cancer patients (11, 12). By integrating medical history, physical signs, and clinical symptoms, PG-SGA provides a comprehensive overview of patients’ nutritional status and functional impairments, enabling clinicians to implement appropriate nutritional interventions during treatment. Lung cancer patients, due to abnormal disease metabolism and treatment side effects, often experience significant weight loss, muscle wasting, and physical decline (13). Therefore, nutritional assessment plays a pivotal role in cancer management. Our study aims to explore the relationships between nutritional status, functional impairment, and quality of life in lung cancer patients. Specifically, it examines the associations of PG-SGA scores with blood biochemical markers, nutritional parameters, and quality-of-life metrics, shedding light on the impact of malnutrition and functional decline on patients’ quality of life.

## Materials and methods

### Materials

A single-center, cross-sectional observational study was conducted at the Affiliated Cancer Hospital of Zunyi Medical University, Zunyi City, Guizhou Province, China, from January 1, 2016, to January 1, 2019. A total of 270 hospitalized lung cancer patients were enrolled. Inclusion criteria included patients aged 18–80 years, fully conscious, without communication barriers, and able to cooperate with examinations. Participants also needed a histological diagnosis of lung cancer, complete medical history records, and follow-up data. Voluntary participation by both patients and their families was required. Exclusion criteria consisted of patients with AIDS or who had undergone organ transplantation, those in critical condition where accurate assessment was infeasible, and those who refused to participate or were unwilling to cooperate with the questionnaire. The study was approved by the ethics committees of all participating institutions and adhered to the principles of the Declaration of Helsinki.

### Assessment method

The Nutritional Risk Screening 2002 (NRS-2002) is a standardized tool recommended by the European Society for Clinical Nutrition and Metabolism (ESPEN) for assessing nutritional risk in hospitalized patients (14, 15). It systematically evaluates three key factors: nutritional status, considering recent weight loss, body mass index (BMI), and dietary intake reduction; disease severity, accounting for increased metabolic demands due to acute or chronic illness; and age, with patients aged ≥70 years receiving an additional risk point. The total score ranges from 0 to 7, with a score of ≥3 indicating nutritional risk and the need for nutritional intervention. By facilitating early identification of at-risk patients, NRS-2002 plays a crucial role in guiding nutritional support strategies, improving clinical outcomes, and optimizing patient care, particularly in hospital and critical care settings.

Patient-Generated Subjective Global Assessment (PG-SGA) is a nutritional assessment tool specifically developed by Ottery for cancer patients (16). It integrates both patient self-reporting and clinical evaluation to comprehensively assess the nutritional status and identify potential risks of malnutrition in cancer patients. The tool consists of two components: self-assessment and clinical assessment. Self-assessment includes four domains—weight, food intake, symptoms, and physical function—evaluating changes over specified periods and symptoms affecting eating. Clinical assessment involves the relationship between disease and nutritional needs, metabolic requirements, and physical examination, focusing on fat stores, muscle condition, and edema. The total score from these components categorizes patients into different risk levels: A, good nutritional status (0–1 points), B, possible malnutrition (2–3 points), C, moderate malnutrition (4–8 points), and severe malnutrition (≥9 points). For patients scoring 4–8, dietary intervention by a nutritionist and further clinical symptom assessment is recommended, while those scoring ≥9 require symptom management and nutritional intervention prior to cancer treatment initiation, reflecting more severe nutritional deficiencies in the patient population. For patients receiving total parenteral or enteral nutrition, PG-SGA scoring was adapted to reflect their artificial nutrition regimen. The food intake component was scored based on feeding tolerance rather than oral intake, considering factors such as gastrointestinal symptoms and metabolic complications. Weight assessment accounted for potential fluid retention. These modifications ensured an accurate evaluation of nutritional status in patients with artificial nutrition support.

The EORTC QLQ-C30 (Quality of Life Core Questionnaire) is a tool developed by the European Organization for Research and Treatment of Cancer (EORTC) to assess the quality of life in cancer patients. It is widely used in clinical research and treatment outcome evaluations for its reliability and validity. The QLQ-C30 covers 15 different domains, including 5 functional domains, 9 symptom domains, and 1 global quality of life domain. Each item is scored on a Likert scale with 4 levels (not at all, a little, quite a bit, very much), scored from 1 to 4, except for items 29 and 30, which are scored on a 7-point scale from very poor to very good (1–7). The global quality of life score is calculated using weighted averages and linear transformation, with a range from 0 to 100. High scores in the functional and global quality of life domains indicate better quality of life, while low scores reflect poorer quality. Conversely, high scores in the symptom domains suggest a higher burden of symptoms, and low scores indicate fewer symptoms.

Patients stood upright in the center of the scale with their arms extended laterally, barefoot, and dressed in light clothing. Body Mass Index (BMI) was calculated using the formula: BMI (kg/m^2^) = weight (kg)/height (m)^2^, based on the measurements of weight (W) and height (H) (17). Measurements of mid-arm circumference (MAC) and triceps skin-fold thickness (TSF) were performed on the non-dominant arm according to Leonard (18). Hand-grip strength (HGS) measurements were conducted in triplicate, with the final value being the average of these three measurements (19). Fasting blood samples were collected within 24 h of admission to assess the levels of white blood cells, neutrophils, lymphocytes, red blood cells, hemoglobin, platelets, serum albumin, prealbumin, total protein, blood urea nitrogen, transaminases, bilirubin, and creatinine. Laboratory data were measured using standard laboratory testing methods.

### Statistical analysis

All statistical analyses were conducted using SPSS version 29.0. Descriptive statistics were employed to analyze the general patient demographics, results from nutritional assessments, quality of life scores, and laboratory test data, including means, standard deviations, frequencies, and percentages. For normally distributed continuous data, means and standard deviations were used; non-normally distributed data were expressed as median (Q25, Q75) and analyzed using non-parametric tests. Correlation analysis was performed using Spearman’s rank correlation to examine the relationship between PG-SGA screening results, quality of life scores, and laboratory test data. Multiple regression analysis was conducted with quality-of-life scores as the dependent variable and PG-SGA screening results as the independent variable, aiming to identify factors influencing the quality of life in lung cancer patients.

## Results

### Demographic and clinical characteristics of the study population

A total of 270 hospitalized lung cancer patients were analyzed, with a majority being male (74.81%) and over 65 years old (80.74%). The population was predominantly Han Chinese (87.78%). The smoking rate was high (73.33%), while the proportion of alcohol consumers was relatively low (22.96%). Among the patients, 51.11% had a habit of drinking tea, and their residential areas were mostly urban and rural (44.45 and 32.59%, respectively). The education level was predominantly high school (56.67%), and most patients were covered by rural cooperative medical insurance (65.56%). The majority of patients were diagnosed at an advanced stage (stage III-IV, 92.59%). Additionally, 90% of patients had no family history, 70.37% had not received radiotherapy or chemotherapy, and 34.44% had chronic diseases. These data highlight the high-risk characteristics and treatment status of lung cancer patients (Table 1).

**Table 1:** The characteristics of lung cancer patients.

Variables	N%
Age (years)	
≤65	52 (19.26)
>65	218 (80.74)
Gender	
Female	68 (25.19)
Male	202 (74.81)
Nationality	
Han	237 (87.78)
Ethnic minority	33 (12.22)
Smoking	
Yes	198 (73.33)
No	72 (26.67)
Alcohol consumption	
Yes	62(22.96)
No	208(77.04)
Tea consumption	
Yes	138(51.11)
No	132(48.89)
Residence	
City	120(44.45)
Town	62(22.96)
Village	88(32.59)
Education	
BS or above	16(5.92)
High school	153(56.67)
Primary school or no schooling	101 (37.41)
Medical insurance	
Urban medical insurance	93 (34.44)
Rural cooperative medical insurance	177 (65.56)
Stage	
I-II	20 (7.41)
III-IV	250 (92.59)
Family history	
Yes	27 (10.00)
No	243 (90.00)
Radiotherapy or chemotherapy	
Yes	80 (29.63)
No	190 (70.37)
Complicated with chronic diseases	
Yes	93 (34.44)
No	177 (65.56)

### Nutritional risk and functional status in lung cancer patients

In this study, Table 2 presents the results of nutritional risk screening (NRS2002) for lung cancer patients. The data show that among patients with NRS2002 scores ≥3 (high nutritional risk group), significantly more patients did not receive nutritional support (66 vs. 41, χ^2^ = 23.7, *p* = 0.001), indicating that patients at higher nutritional risk are more likely to lack nutritional intervention. Table 3 displays the results of PG-SGA and Karnofsky scores. The PG-SGA results show that the majority of patients fall into category C (malnourished, 105 patients), followed by category A (no change in nutritional status, 100 patients) and category B (at risk of malnutrition, 65 patients), suggesting that malnutrition is prevalent among lung cancer patients. Additionally, the Karnofsky scores indicate that most patients have a good functional status (score ≥ 70, 258 patients), though a small proportion of patients have severe functional impairment (score < 70, 12 patients). There are no significant gender differences in the PG-SGA and Karnofsky scores (*p*-values of 0.147 and 0.574, respectively), indicating that gender has little impact on nutritional risk and functional status in this sample of lung cancer patients.

**Table 2:** Results of nutritional risk screening for lung cancer patients(n).

NRS2002	n	Nutritional support	No nutritional support	χ^2^	*P*
≥3	107	41	66	23.7	0.001
<3	163	21	142		

**Table 3:** PG-SGA and Karnofsky scores of lung cancer patients.

Scores	n	Mean ± standard deviation	Males	Females	*p*
PG-SGA	270	9.14 ± 7.11			0.147
A(0–3)	100		81	19	
B(4–8)	65		51	14	
C(≥9)	105		75	30	
Karnofsky	270	84.85 ± 10.89			0.574
≥70	258		192	66	
<70	12		10	2	

A, no change in nutritional status; B, at risk of malnutrition and C, malnourished.

### Nutritional support utilization in lung cancer patients: a gender and risk-based analysis

Among the 270 lung cancer patients, 208 (77.04%) did not receive any form of nutritional support. This included 153 males (73.56% of the total male population) and 55 females (26.44% of the total female population). Among patients with a PG-SGA score ≥ 4, 115 patients (67.65%) did not receive any nutritional support, with 78 males (45.88% of the male PG-SGA ≥4 group) and 37 females (21.76% of the female PG-SGA ≥4 group). For those who did receive nutritional support, the distribution was as follows: 46 patients (17.04%) received only parenteral nutrition (PN), including 36 males (78.26%) and 10 females (21.74%). Among the PG-SGA ≥4 group, this was 25.29%, with 33 males (76.74%) and 10 females (23.26%). Only 16 patients (5.93%) received enteral nutrition (EN), including 12 males (75.00%) and 4 females (25.00%). In the PG-SGA ≥4 group, this was 8.24%, with 10 males (71.43%) and 4 females (28.57%). Eight patients (2.96%) received both EN and PN support, including 6 males (75.00%) and 2 females (25.00%). In the PG-SGA ≥4 group, this was 4.71%, with equal gender representation (75.00%). This demonstrates that the use of nutritional support among lung cancer patients is low, particularly among those at higher nutritional risk (Table 4).

**Table 4:** The nutritional support in lung cancer patients.

Nutrition support	Total (%)	Male (%)	Female (%)	PG-SGA ≥ 4		
				Total (%)	Male (%)	Female (%)
No	208 (77.04)	153 (73.56)	55 (26.44)	115 (67.65)	78 (45.88)	37 (21.76)
Yes						
PN	46 (17.04)	36 (78.26)	10 (21.74)	43 (25.29)	33 (76.74)	10 (23.26)
EN	16 (5.93)	12 (75.00)	4 (25.00)	14 (8.24)	10 (71.43)	4 (28.57)
EN and PN	8 (2.96)	6 (75.00)	2 (25.00)	8 (4.71)	6 (75.00)	2 (75.00)

PN, parenteral nutrition; EN, enteral nutrition.

### Correlation between PG-SGA scores and biochemical markers in lung cancer patient

This study demonstrated significant associations between PG-SGA scores and various blood biochemical indicators in lung cancer patients. As the PG-SGA score increased, serum prealbumin (PA) levels significantly decreased (PA: 197.85 ± 7.42 in the PG-SGA ≥9 group vs. 223.76 ± 66.07 in the 0–3 group, *p* = 0.030). Total bilirubin (TBIL) levels were significantly elevated in higher PG-SGA score groups (11.02 ± 5.25 in the ≥9 group vs. 8.79 ± 3.72 in the 0–3 group, *p* = 0.000). Alanine aminotransferase (ALT) decreased significantly as PG-SGA scores increased (23.49 ± 15.77 in the ≥9 group vs. 37.75 ± 52.21 in the 0–3 group, *p* = 0.033). Lymphocyte count (LYMPH) was lower in higher PG-SGA score groups (1.16 ± 0.58 in the ≥9 group vs. 1.43 ± 0.97 in the 0–3 group, *p* = 0.027). Additionally, the PG-SGA score was positively correlated with the white blood cell/red blood cell ratio (WBC/RBC) (1.73 ± 0.94 in the ≥9 group vs. 1.45 ± 0.42 in the 0–3 group, *p* = 0.030). The total bilirubin/direct bilirubin ratio (TBIL/DBIL) was also elevated in patients with higher PG-SGA scores (4.91 ± 2.85 in the ≥9 group vs. 3.70 ± 1.02 in the 0–3 group, *p* = 0.000). These results suggest that poorer PG-SGA scores correlate with nutritional deficiencies and metabolic disturbances in lung cancer patients. Indicators such as prealbumin, total bilirubin, alanine aminotransferase, and lymphocyte count are critical for assessing the nutritional status of lung cancer patients (Table 5).

**Table 5:** Association between the PG-SGA and blood biochemistry results.

Parameters	PG-SGA			*F*	*P*
	A (0–3)	B (4–8)	C(≥9)		
TP	65.87 ± 9.08	64.75 ± 6.53	65.77 ± 7.21	0.953	0.387
Cr	74.74 ± 17.02	72.54 ± 15.76	72.15 ± 16.59	0.693	0.501
ALB	38.08 ± 6.83	36.50 ± 4.43	37.21 ± 5.63	1.615	0.201
Urea	4.73 ± 2.19	4.59 ± 2.84	4.55 ± 2.11	0.154	0.857
PA	223.76 ± 66.07	213.67 ± 65.69	197.85 ± 7.42	3.544	0.030
TBIL	8.79 ± 3.72	8.63 ± 3.17	11.02 ± 5.25	9.192	0.000
DBIL	2.82 ± 2.63	2.58 ± 1.46	3.18 ± 2.07	1.694	0.186
GLU	4.98 ± 0.72	5.43 ± 1.57	5.13 ± 0.90	2.533	0.082
TG	1.27 ± 0.70	2.19 ± 2.57	1.99 ± 1.51	0.578	0.569
AST	26.02 ± 12.77	28.94 ± 19.29	27.21 ± 13.89	0.743	0.477
ALT	37.75 ± 52.21	31.72 ± 37.99	23.49 ± 15.77	3.460	0.033
HGB	120.00 ± 16.80	117.37 ± 23.98	116.91 ± 20.21	0.678	0.050
WBC	5.86 ± 2.37	7.00 ± 4.02	6.52 ± 3.12	2.828	0.061
NEUT	3.88 ± 2.26	4.06 ± 2.36	4.55 ± 2.87	1.881	0.155
LYMPH	1.43 ± 0.97	1.44 ± 0.81	1.16 ± 0.58	3.645	0.027
RBC	4.14 ± 0.80	3.96 ± 0.69	3.91 ± 0.66	2.615	0.075
PLT	232.27 ± 112.06	234.34 ± 105.99	248 ± 87.34	0.726	0.485
NEUT/WBC	0.64 ± 0.20	0.66 ± 0.14	0.65 ± 0.18	0.967	0.381
WBC/RBC	1.45 ± 0.42	1.79 ± 1.15	1.73 ± 0.94	3.560	0.030
TBIL/DBIL	3.70 ± 1.02	3.78 ± 1.38	4.91 ± 2.85	10.870	0.000
PA/ALB	5.58 ± 1.90	5.52 ± 2.08	4.96 ± 1.38	1.685	0.187
PA/TB	3.38 ± 1.10	3.19 ± 1.22	2.90 ± 1.10	2.945	0.054

A, no change in nutritional status; B, at risk of malnutrition and C, malnourished.

### Impact of PG-SGA scores on nutritional parameters and body composition in lung cancer patients

We investigated the association between PG-SGA scores and various nutritional parameters, finding significant differences across different PG-SGA score groups (*p* < 0.05). NRS2002 scores, KPS scores, and mid-arm circumference (MAC) decreased with increasing PG-SGA scores, with significant differences observed for both NRS2002 and KPS scores (*p* < 0.0001) and MAC (*p* < 0.0001). Body weight (BW) and triceps skinfold thickness (TSF) also showed significant differences (*p* = 0.029 and *p* < 0.0001, respectively). Handgrip strength (HGS) was significantly higher in the PG-SGA 4–8 group compared to the ≥9 group (*p* < 0.0001). Mid-arm muscle circumference (MAMC) did not show significant differences between groups (*p* = 0.107). Additionally, the maximum circumference of the calves (MCC) decreased with increasing PG-SGA scores, with significant differences observed (*p* < 0.0001). These results suggest that with worsening nutritional status (higher PG-SGA scores), a decline in various nutritional parameters, particularly those related to body composition and functional capacity, was observed (Table 6).

**Table 6:** Association between the PG-SGA and nutritional parameters results.

Parameters	PG-SGA			F	*p*
	0–3	4–8	≥9		
NRS2002 (score)	1.37 ± 0.75	2.43 ± 1.21	3.24 ± 1.28	90.556	0.000
KPS (score)	90.60 ± 5.83	85.85 ± 6.82	78.76 ± 13.28	39.283	0.000
BW (Kg)	61.26 ± 9.9	57.41 ± 9.18	58.51 ± 9.86	3.598	0.029
BMI (kg/m^4^)	22.87 ± 2.96	21.88 ± 2.78	22.06 ± 3.13	2.849	0.060
MAC (cm)	28.34 ± 4.65	25.92 ± 4.24	24.19 ± 2.33	30.747	0.000
TSF (mm)	23.36 ± 17.93	13.63 ± 11.56	6.75 ± 4.39	44.777	0.000
HGS (kg)	22.87 ± 9.40	24.42 ± 8.86	22.11 ± 8.39	15.126	0.000
MAMC (cm)	21.01 ± 4.78	21.64 ± 3.20	22.07 ± 3.61	2.253	0.107
MCC-right (cm)	33.50 ± 3.33	32.40 ± 2.91	31.75 ± 3.19	8.270	0.000
MCC-left (cm)	33.52 ± 3.27	32.41 ± 3.09	31.77 ± 2.88	8.415	0.000

BMI, body mass index; MAC, mid-arm diameter; TSF, triceps skin-fold; HGS, hand-grip strength; MAMC, Mid-Arm Muscle Circumference; MCC, Maximum calf circumference.

### Impact of nutritional status on quality of life in lung cancer patients: a PG-SGA analysis

This study explored the relationship between nutritional status, assessed by PG-SGA, and quality of life (QOL) in lung cancer patients. Higher PG-SGA scores were significantly associated with declines in physical, role, emotional, and social function (e.g., physical function dropped from 93.33 in the 0–3 group to 40.00 in the ≥9 group, *p* < 0.0001). Symptoms like fatigue, nausea/vomiting, pain, insomnia, and loss of appetite worsened with increasing PG-SGA scores (e.g., fatigue increased from 32.69 in the 0–3 group to 49.69 in the 4–8 group, *p* < 0.0001). Economic issues also rose (*p* = 0.001), while diarrhea showed no significant differences (*p* = 0.978) (Tables 7). Correlation analysis revealed that PG-SGA scores were positively correlated with functional impairments (e.g., physical function, *r* = 0.605, *p* < 0.001) and symptoms like fatigue (*r* = 0.667, *p* < 0.001). Conversely, PG-SGA scores negatively correlated with overall QOL (*r* = −0.687, *p* < 0.001). No significant correlations were found for shortness of breath, appetite loss, constipation, diarrhea, or economic issues. These findings highlight the significant impact of poor nutritional status on QOL in lung cancer patients (Table 8).

**Table 7:** The correlation between nutritional status and quality of Life in lung cancer patients [Q50(Q25, Q75)].

Categories	PG-SGA			χ^2^	p*
	0–3	4–8	≥9		
Physical functioning	93.33 (86.67, 93.33)	84.44 (77.78, 86.67)	40.00 (20.00, 70.00)	149.07	0.000
Role functioning	89.20 (74.07, 100)	68.98 (52.60, 84.16)	54.84 (31.09, 83.63)	59.16	0.000
Emotional functioning	94.56 (85.15, 100)	87.88 (72.82, 95.84)	77.38 (64.84, 91.54)	48.98	0.000
Cognitive functioning	81.36 (70.40, 92.69)	71.57 (52.78, 87.90)	83.94 (39.37, 83.94)	30.89	0.000
Social functioning	57.78 (40.48, 76.52)	38.61 (25.43, 53.43)	50.75 (34.12, 69.82)	32.28	0.000
Global QOL	87.32 (74.74, 100)	73.21 (55.36, 85.63)	48.99 (35.00, 65.00)	113.69	0.000
Fatigue	32.69 (23.38, 43.92)	49.69 (34.21, 61.91)	18.71 (8.45, 29.33)	95.21	0.000
Nausea/Vomiting	7.61 (00.00, 34.78)	5.00 (00.00, 14.03)	25.88 (6.33, 57.89)	62.36	0.000
Pain	11.31 (00.00, 27.86)	21.49 (6.13, 36.73)	32.35 (14.70, 50.86)	40.41	0.000
Dyspnea	1.83 (1.17, 2.59)	17.26 (00.00, 40.12)	21.59 (1.70, 47.11)	11.91	0.003
Insomnia	16.67 (00.00, 36.29)	28.98 (5.43, 59.32)	45.40 (13.52, 76.60)	31.25	0.000
Appetite loss	24.00 (00.00, 74.00)	21.11 (3.06, 43.05)	56.41 (27.66, 85.31)	56.54	0.000
Constipation	9.09 (00.00, 34.34)	7.53 (00.00, 25.00)	25.78 (2.44, 61.51)	38.17	0.000
Diarrhea	4.66 (00.00, 22.58)	4.23 (00.00, 21.43)	4.42 (00.00, 22.28)	0.05	0.978
Financial problems	40.82 (19.05, 59.55)	40.10 (21.25, 71.34)	50.03 (33.00, 76.67)	14.32	0.001

*Kruskal-Wallis tests, *p* < 0.01.

**Table 8:** Correlation analysis between PG-SGA quantitative evaluation and quality of life in patients with lung cancer.

EORTC QLQ-C30	Correlation coefficient*	*P*
Functional scales		
Physical functioning	0.605	0.000
Role functioning	0.662	0.000
Emotional functioning	0.535	0.000
Cognitive functioning	0.379	0.000
Social functioning	0.551	0.000
Global QOL	−0.687	0.000
Symptom scales		
Fatigue	0.667	0.000
Nausea/vomiting	0.584	0.000
Pain	0.498	0.000
Dyspnea	−0.059	0.332
Insomnia	0.364	0.000
Appetite loss	−0.007	0.915
Constipation	−0.016	0.798
Diarrhea	0.008	0.891
Financial problems	0.011	0.855

*Spearman rank correlation coefficient, *p* < 0.05.

### Univariate analysis of nutritional status

The results of the univariate binary Logistic regression analysis for severe malnutrition show that there are significant associations between severe malnutrition and alcohol consumption (*p* = 0.009) as well as tea consumption (*p* = 0.001). Non-drinkers have a 2.149-fold higher risk of developing severe malnutrition compared to drinkers (OR = 2.149, 95% CI: 1.210–3.819), and non-tea drinkers have a 2.334-fold higher risk compared to tea drinkers (OR = 2.334, 95% CI: 1.411–3.862). However, the *p* values of variables such as gender, age, nationality, smoking, disease stage, family history, and BMI are all greater than 0.05, indicating no statistical significance (Table 9).

**Table 9:** Univariate analysis of severe malnutrition.

Categories	*P*	OR (95%CI)
Gender	0.307	
Female		1
Male		1.337 (0.766 ~ 2.334)
Age	0.453	
<65		1
≥65		1.262 (0.687 ~ 2.320)
Nationality	0.116	
Han		1
Ethnic minority		0.556 (0.267 ~ 1.156)
Smoking	0.573	
Yes		1
No		0.854 (0.493 ~ 1.479)
Alcohol consumption	0.009	
Yes		1
No		2.149 (1.210 ~ 3.819)
Stage	0.066	
I–II		1
III–IV		0.532 (0.272 ~ 1.041)
Family history	0.301	
Yes		1
No		1.524 (0.686 ~ 3.386)
BMI (kg/m^2^)	0.256	0.953 (0.878 ~ 1.035)
Tea consumption	0.001	
Yes		1
No		2.334 (1.411 ~ 3.862)

## Discussion

This study analyzed the nutritional status, functional status, and quality of life (QOL) in 270 hospitalized lung cancer patients and investigated the relationship between PG-SGA scores and patient quality of life and nutritional parameters. Our data highlighted the high-risk characteristics of lung cancer patients, underscoring the importance of nutritional support in clinical treatment. Through analysis of patient demographic characteristics, we found that most patients were elderly males with a high smoking rate, reflecting the typical high-risk profile for lung cancer (20, 21). These findings are consistent with other similar studies, highlighting that smoking and aging are significant risk factors for lung cancer (22, 23). Furthermore, the majority of patients were diagnosed at an advanced stage (III-IV), further corroborating the commonality of late-stage diagnosis (24). Regarding nutritional status, although PG-SGA scores indicated that most patients experienced some degree of malnutrition, the majority of patients did not receive nutritional support (77.04%). This was particularly evident in the group with higher nutritional risk (PG-SGA ≥4), where 67.65% of patients did not receive any form of nutritional support. This highlights the insufficient use of nutritional intervention in lung cancer treatment, especially among those at high nutritional risk (25). Previous research has shown that malnutrition is linked to adverse clinical outcomes in cancer patients, emphasizing the crucial role of timely nutritional intervention in improving treatment outcomes and quality of life.

Moreover, an important finding of this study is the relationship between PG-SGA scores and various biochemical indicators. Patients with higher PG-SGA scores exhibited significant differences in prealbumin (PA), total bilirubin (TBIL), alanine transaminase (ALT), and lymphocyte count (LYMPH), indicating that PG-SGA scores can effectively link nutritional status with biochemical parameters. This is consistent with previous research, suggesting that PG-SGA scores not only reflect clinical nutritional status but also closely correlate with physiological indicators (26). The significant correlation between PG-SGA scores and multiple nutritional parameters, such as NRS2002 scores, KPS scores, mid-arm circumference, and body weight, further supports the validity of PG-SGA in assessing the nutritional status of lung cancer patients (27, 28). As PG-SGA scores increase, there is a noticeable decline in indicators related to body composition and functional capacity, particularly in changes observed in KPS scores and mid-arm circumference (29). Studies have shown that malnutrition directly impacts physical strength and functional status, which subsequently affects quality of life. Another important finding of this study is the significant association between PG-SGA scores and quality of life in lung cancer patients. As PG-SGA scores rise, there is a significant decrease in scores across multiple dimensions, including physical functioning, role functioning, emotional functioning, and social functioning. Symptoms such as fatigue, nausea/vomiting, pain, breathing difficulties, insomnia, and loss of appetite worsen with higher PG-SGA scores. These results align with existing research, demonstrating that malnutrition not only negatively affects patient functional status but also exacerbates the manifestation of cancer-related symptoms, significantly reducing quality of life (30, 31). Particularly, the significant decline in physical and role functioning suggests that, during lung cancer treatment, greater attention should be paid to patients’ physical health and daily living capabilities, and personalized nutritional interventions should be implemented to alleviate these symptoms.

Lastly, although PG-SGA scores did not show significant correlations with symptoms such as diarrhea, loss of appetite, and economic issues, their potential impact should not be overlooked. Symptoms like diarrhea are common among cancer patients, potentially linked to the medications used or the progression of the disease itself (32). Regarding economic issues, despite the lack of significant correlation, their prevalence among lung cancer patients highlights the financial stress patients may face during treatment, particularly in the absence of adequate insurance support (33).

Further, findings from the univariate binary Logistic regression analysis for severe malnutrition deserve attention. It showed significant associations between severe malnutrition and alcohol consumption (*p* = 0.009), as well as tea consumption (*p* = 0.001). Specifically, non-drinkers had a 2.149-fold higher risk of severe malnutrition than drinkers (OR = 2.149, 95% CI: 1.210–3.819), and non-tea drinkers faced a 2.334-fold higher risk compared to tea drinkers (OR = 2.334, 95% CI: 1.411–3.862).

While this study revealed significant associations between the nutritional status and quality of life in lung cancer patients, it also had several limitations. First, as a cross-sectional study, it cannot establish causality. Second, the sample was drawn from a single medical institution, which may limit the generalizability of the results. Third, the PG-SGA score might be subject to assessor bias, and it did not fully account for all potential influencing factors, such as psychosocial elements and medication use during treatment. The study also did not delve deeply into the specific impact of different types of nutritional interventions on patient quality of life. Future multi-center, longitudinal studies will help to further validate these associations and refine nutritional intervention strategies. Additionally, future prospective studies could further explore the association between nutritional status, quality of life, and survival outcomes in lung cancer patients.

Overall, this study underscores the importance of nutritional assessment and intervention in lung cancer treatment, particularly in patients at high nutritional risk. The association between PG-SGA and various parameters highlights its potential as a valuable tool for assessing nutritional status and predicting quality of life. Future research should focus on developing targeted interventions to improve nutritional support and ultimately enhance the quality of life and survival outcomes for lung cancer patients. Additionally, further exploration of the potential mechanisms linking nutritional status with disease progression is necessary.

## Data Availability

The original contributions presented in the study are included in the article/supplementary material, further inquiries can be directed to the corresponding author.
